# CD8^+^ T cell depletion prevents neuropathology in a mouse model of globoid cell leukodystrophy

**DOI:** 10.1084/jem.20221862

**Published:** 2023-06-13

**Authors:** Pearl A. Sutter, Antoine Ménoret, Evan R. Jellison, Alexandra M. Nicaise, Allison M. Bradbury, Anthony T. Vella, Ernesto R. Bongarzone, Stephen J. Crocker

**Affiliations:** 1 Department of Neuroscience, University of Connecticut School of Medicine, Farmington, CT, USA; 2 Department of Immunology, University of Connecticut School of Medicine, Farmington, CT, USA; 3 Department of Clinical Neuroscience and National Institute for Health Research Biomedical Research Centre, University of Cambridge, Cambridge, UK; 4 Department of Pediatrics, Nationwide Children's Hospital, Ohio State University, Columbus, OH, USA; 5 Department of Anatomy and Cell Biology, University of Illinois at Chicago, Chicago, IL, USA

## Abstract

Globoid cell leukodystrophy (GLD) or Krabbe’s disease is a fatal genetic demyelinating disease of the central nervous system caused by loss-of-function mutations in the galactosylceramidase (galc) gene. While the metabolic basis for disease is known, the understanding of how this results in neuropathology is not well understood. Herein, we report that the rapid and protracted elevation of CD8^+^ cytotoxic T lymphocytes occurs coincident with clinical disease in a mouse model of GLD. Administration of a function-blocking antibody against CD8α effectively prevented disease onset, reduced morbidity and mortality, and prevented CNS demyelination in mice. These data indicate that subsequent to the genetic cause of disease, neuropathology is driven by pathogenic CD8^+^ T cells, thus offering novel therapeutic potential for treatment of GLD.

## Teaser

Globoid cell leukodystrophy (GLD) is an incurable genetic disease characterized by profound white matter loss and inflammation. This report identifies CD8^+^ T cells as a novel pathogenic process in the clinical development and neuropathology of this disease.

## Introduction

Globoid cell leukodystrophy (GLD) is a fatal demyelinating disease of the central nervous system (CNS) that leads to paralysis and death in 99% of affected children before the age of 5 (Barczykowski et al., 2012; Wenger and Luzi, 2020). Loss-of-function mutation in the enzyme galactocerebrosidase (GALC) in GLD leads to an accumulation of galactosylsphingosine (“psychosine”), which is known to contribute to the hallmarks of GLD neuropathology, including neuroinflammation and demyelination (Santambrogio et al., 2012; Potter et al., 2013). Early hypotheses suggested that the observed oligodendrocyte death and demyelination in GLD was a direct result of psychosine accumulation; however, more recent findings have shown that demyelination in GLD is temporally preceded by neuroinflammation (Nicaise.et al., 2016; Potter and Petryniak, 2016; Feltri et al., 2021). These findings suggest that the build-up of psychosine to supraphysiological levels may initiate or promote initiation of CNS inflammation, which is culpable for mediating neuropathology and the resulting progressive physical decline and demyelination.

CNS inflammation in GLD is characterized by the presence of astrogliosis and microgliosis. The presence of activated astrocytes, macrophages, and microglia have been well established as preceding oligodendrocyte cell death in GLD; however, their roles in disease are largely unresolved. While the prevalence of activated macrophages and microglia has been found to lessen as the disease becomes fulminant (Ohno et al., 1993; Itoh et al., 2002), early studies on the CNS of twitcher (twi) mice, a murine model of GLD, also reported the presence of T cells at end-stage disease (Ohno et al., 1993; Matsushima et al., 1994; Suzuki and Taniike, 1995; Taniike et al., 1997). These findings led to a study testing the role of CD4^+^ T cells in GLD pathology where MHC II expression was globally eliminated. Elimination of MHC II in twi mice, which resulted in a decrease in CD4^+^ T cell populations, was found to reduce macrophage/microglia activation and delayed onset of disease signs but did not extend survival (Wu et al., 2001). Additional studies found that CD8^+^ T cells were found to be more plentiful than CD4^+^ T cells in demyelinated areas of the twi mouse brain, leading to a study where expression of MHC I was examined and found to increase as twi disease progressed (Taniike et al., 1997). However, the functional role of CD8^+^ T cells was not examined in this study. Several additional histological studies have also identified the presence of increased CD8^+^ T cells in human GLD CNS (Itoh et al., 2002; Iacono et al., 2022), further emphasizing that the role of T cells in GLD should be investigated.

These previous findings suggest there is a role for adaptive immunity and CD8^+^ T cells in GLD; however, a functional role for CD8^+^ T cells had not been identified. Our study characterized the CD8^+^ T cell population in murine GLD through disease progression and investigated the functional role of CD8^+^ T cells in GLD. We found that depletion of CD8^+^ T cells in the twi mouse resulted in profound differences in disease course and pathological outcomes, which has laid a foundation for investigation of the role of adaptive immunity in GLD.

## Results and discussion

### CD8^+^ T cell influx into the CNS correlates with twi disease progression

**Figure 1.**
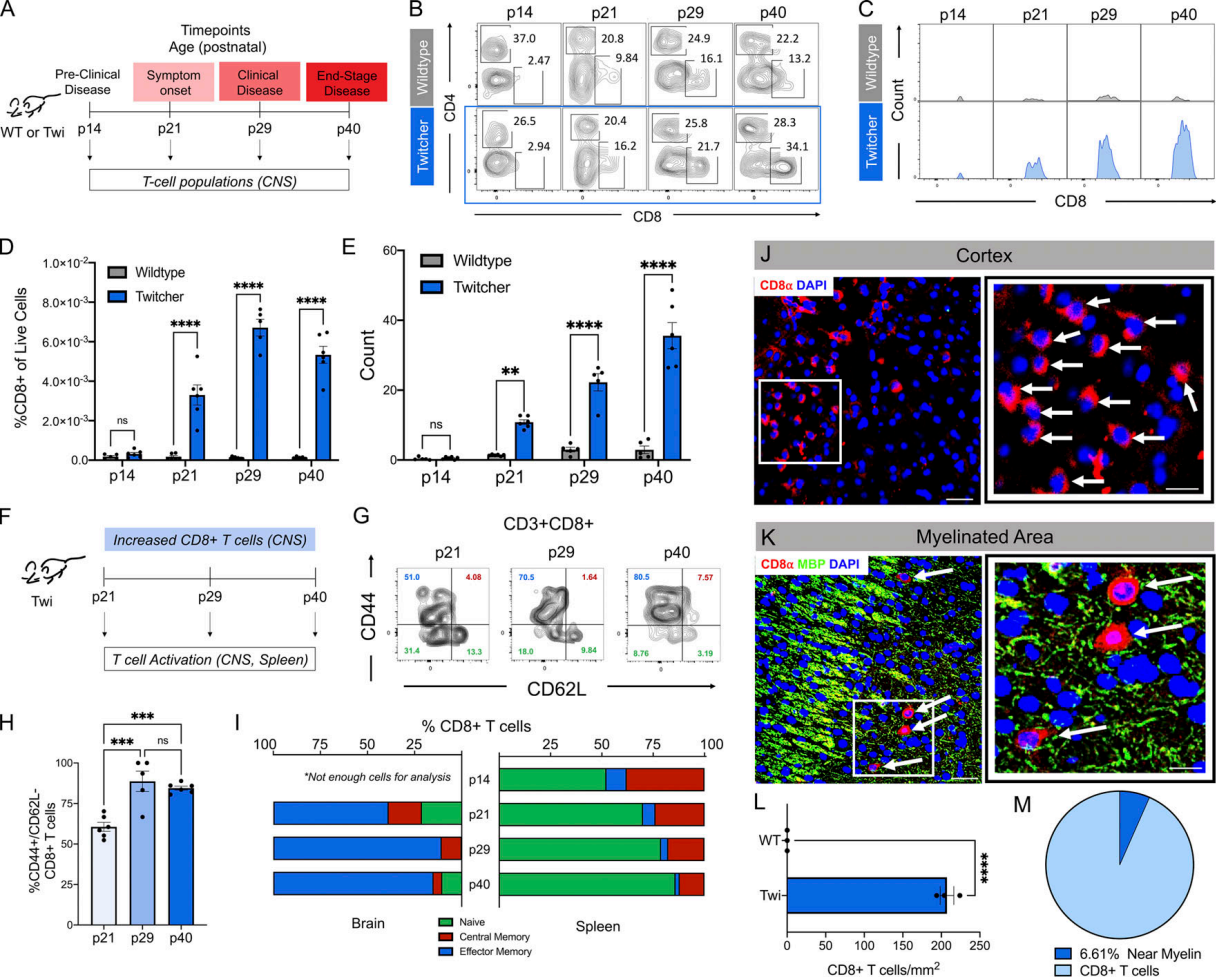
**Robust activated CD8**^**+**^ **T cell populations in the twi CNS correlated with disease progression. (A)** Schematic showing twi disease progression with correlated murine postnatal age. **(B)** Flow cytometry plots showing the percentage of CD4^+^ and CD8^+^ T cells (gated on Scatter:Live:CD45^high^:CD3^+^; Fig. S1 A) in WT and twi mouse brains at four time points during disease progression; three technical replicates/biological replicate averaged, *n* = 5–6 biological replicates, three independent experiments. **(C)** Histograms showing counts of CD8^+^ T cells in WT and twi mice during disease progression; three technical replicates/biological replicate averaged, *n* = 5–6 biological replicates, three independent experiments. **(D)** Percent of CD3^+^:CD8^+^ T cells out of live cells in WT and twi CNS; three technical replicates/biological replicate averaged, *n* = 5–6 biological replicates, three independent experiments. **(E)** Counts of CD8^+^ T cells in WT and twi mice brains, three technical replicates/biological replicate averaged, *n* = 5–6 biological replicates, three independent experiments. **(F)** Schematic showing analysis of CD8^+^ T cell activation was performed in twi CNS and spleens. **(G)** Representative flow plots of gated twi CD8^+^ T cells in twi CNS gated by CD44 and CD62L to characterize T cell activation in CNS. **(H)** The percent of effector memory CD8^+^ T cells in twi CNS increased with disease progression. **(I)** Twi CD8^+^ T cells in CNS vs. spleen show differences in percentages of activated T cells. **(J and K)** Representative image of clustered CD8^+^ T-cells in twi cortex (J) and myelinated areas (K) at p29. White box indicates area for zoomed-in image on right; white arrows point to CD8^+^ T cells; red = CD8, green = MBP, blue = DAPI. **(L and M)** (L) Density of CD8^+^ T cells is increased in twi CNS at p29 with (M) 6.61% of CD8^+^ T cells found in myelinated areas, *n* = 10 images/animal (5 cortex, 5 myelinated areas), 3 animals/group. Scale bars on left (J and K) 50 μm, right (J and K) 20 μm. **P < 0.01, ***P < 0.001, ****P < 0.0001; statistical tests used were *t* tests (L), one-way ANOVA (H), two-way ANOVA (D and E).

**Figure S1.**
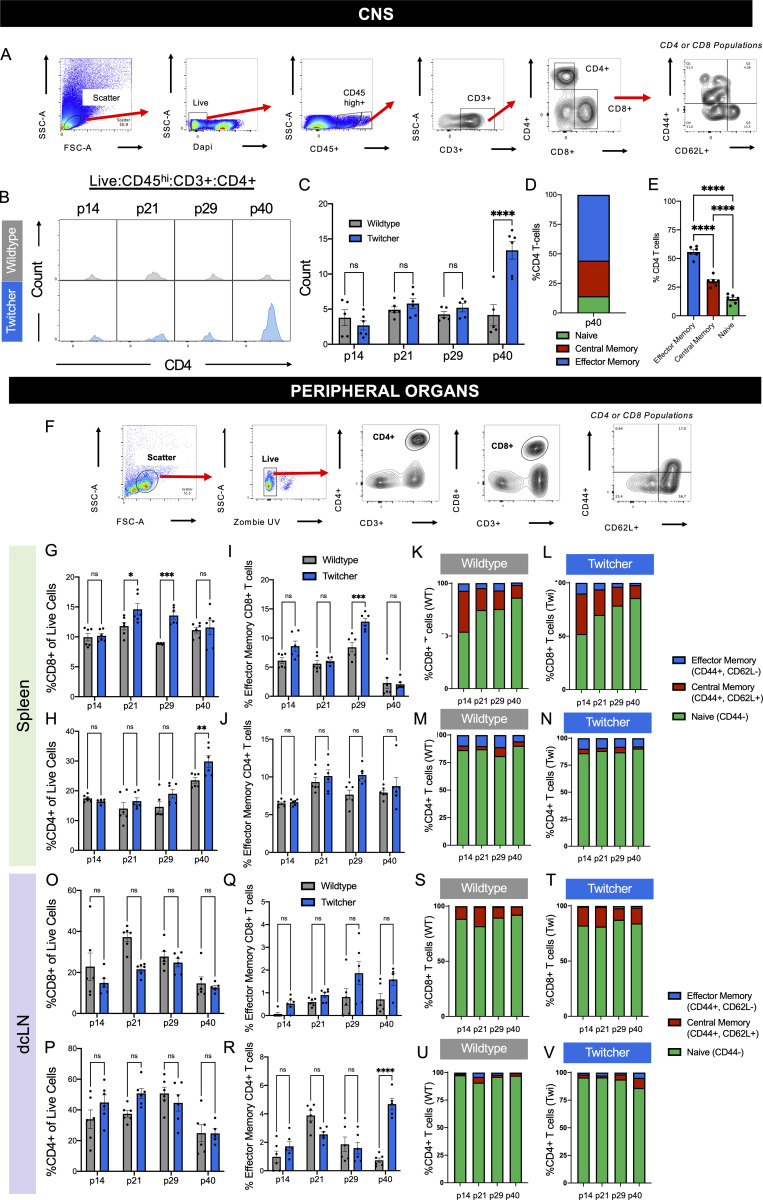
**Flow cytometry analysis of CD4**^**+**^ **T cell populations in twi CNS show influx at p40 with a limited T cell response in twi spleens and dcLNs. (A)** CNS flow cytometry gating strategy for identification and analysis of T cell populations. **(B)** CD4^+^ T cells (gated as seen in A) increase in twi mice at end-stage disease (p40) as shown by representative histograms. **(C)** Count of CD4^+^ T cell population in WT and twi mice; *n* = 5–6 biological replicates, three independent experiments. **(D and E)** Analysis of activation states of twi CD4^+^ T cells shows a majority of the CD4^+^ T cells present in twi CNS at p40 are activated; *n* = 5–6 biological replicates, three independent experiments. **(F)** Flow cytometry gating strategy for identification of T cell populations in spleen and dcLNs. **(G)** Percentages of CD3^+^ CD8^+^ cells in twi spleens showed an increase at p21 and p29 similar to twi brain; *n* = 6 biological replicates, three independent experiments. **(H)** CD3^+^ CD4^+^ cells in spleen did not differ between WT and twi mice during twi disease progression; *n* = 6 biological replicates, three independent experiments. **(I–N)** (I) Percentages of effector memory CD8^+^ or (J) CD4^+^ T cells (CD44^+^/CD62L^−^) showed no differences between WT and twi spleens, *n* = 6 biological replicates, three independent experiments. Characterization of CD8^+^ (K and L) and CD4^+^ (M and N) T cell activation in WT and twi mice show similar patterns for naive (CD44^−^/CD62L^+/−^), central memory (CD44^+^/CD62^+^), and effector memory percentages (CD44^+^/CD62L^−^); *n* = 6 biological replicates, three independent experiments. **(O and P)** Percentages of CD3^+^ CD8^+^ (O) and CD3^+^ CD4^+^ (P) T cells in twi dcLNs did not differ between WT and twi during twi disease progression; *n* = 6 biological replicates, three independent experiments. (Q and R) Percentages of effector memory CD8^+^ (Q) T cells (CD44^+^/CD62L^−^) showed no differences between WT and twi dcLN, however at p40 there was an expansion of CD4^+^ (R) effector memory T cells in twi mice that was similar to what was seen in twi brain; *n* = 6 biological replicates, three independent experiments. **(S–V)** Characterization of CD8^+^ (S and T) and CD4^+^ (U and V) T cell activation in WT and twi mice show similar patterns for naive (CD44^−^/CD62L^+/−^), central memory (CD44^+^/CD62^+^), and effector memory percentages (CD44^+^/CD62L^−^); *n* = 6 biological replicates, three independent experiments. *P < 0.05, ***P < 0.001, ****P < 0.0001; statistical tests used include one-way ANOVA (E) and two-way ANOVA (C, G–J, and O–R).

**Figure S2.**
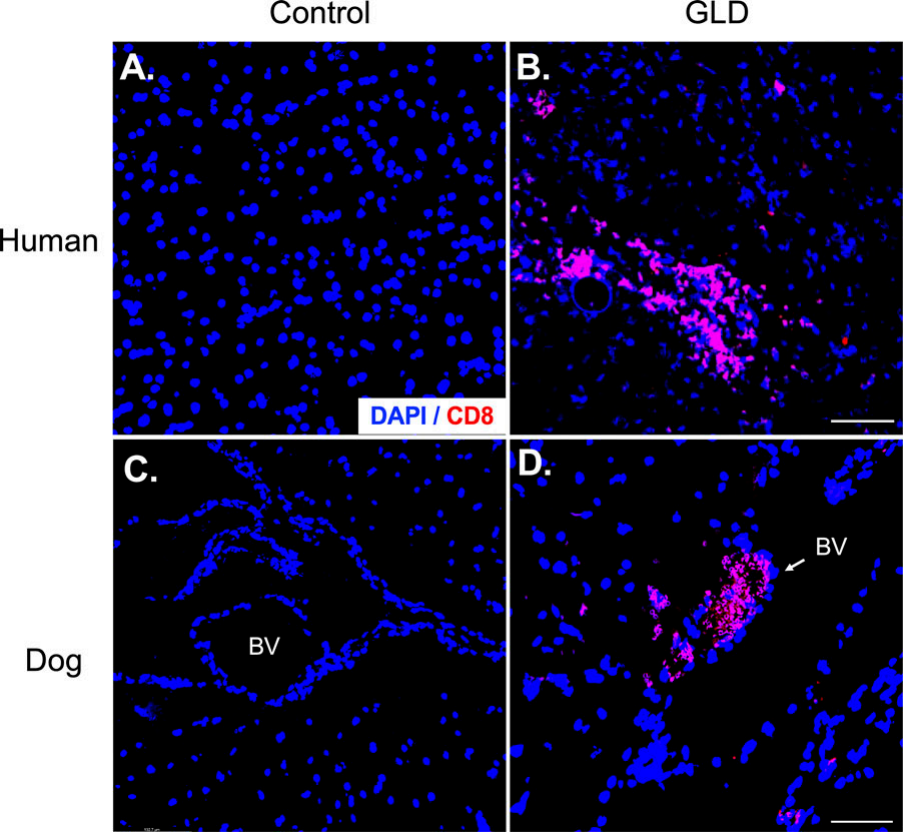
**CD8**^**+**^ **T cells are present in the CNS of dog and human GLD. (A and B)** CD8^+^ T cells clusters are present in CNS white matter of infantile human GLD; *n* = 1/group. **(C and D)** CD8^+^ T cells infiltrating CNS tissue in dog GLD from blood vessels (BV); *n* = 1/group. Scale bars are 100 μm.

To better understand the T cell populations within the CNS of twi mice, we conducted flow cytometry at four time points spanning the short 45-d lifespan of twi mice which coincided with established phases of disease progression: preclinical disease (postnatal [p]14), disease onset (p21), fully developed clinical disease (p30), and end-stage disease (p40; Fig. 1 A and Fig. S1 A). A significant increase in CD8^+^ T cells at the time of disease onset (p21) was identified and found to continue to increase as disease progressed (Fig. 1, B–E). Activated CD4^+^ T cells were also identified in twi CNS at p40, yet their relative numbers were lower than CD8^+^ T cells and CNS infiltration was not observed until the end stage of disease ∼p40 (Fig. S1, B and C). Measurement of effector memory CD8^+^ T cells (CD8^+^/CD44^+^/CD62L^−^) confirmed that the CNS infiltrating CD8^+^ T cells in twi mice were highly activated during twi disease progression (Fig. 1, F–H), as were the infiltrating CD4^+^ T cells at p40 (Fig. S1, D and E). In contrast, comparison of each CD8^+^ and CD4^+^ effector memory (CD44^+^/CD62L^−^) populations from spleens and deep cervical lymph nodes (dcLNs) of twi and WT littermate controls showed similar levels of activation, which suggested negligible differences in CD8^+^ T cell activation outside the CNS (Fig. 1 I and Fig. S1, F–V). Immunohistochemistry (IHC) on twi CNS tissues identified CD8^+^ T cells in cortex (Fig. 1 J) and white matter (myelin basic protein+ [MBP]; Fig. 1 K). A significantly higher number of CD8^+^ T cells were found in twi compared with WT, with a majority of the CD8^+^ T cells being found in the cortex (Fig. 1, L and M). No CD8^+^ T cells were observed in WT littermates (Fig. 1 L). Additionally, we confirmed the presence of CD8^+^ T cells in human GLD brain white matter (Fig. S2, A and B), as well as in naturally occurring canine GLD models in the perivascular space (Fig. S2, C and D).

### Depletion of CD8^+^ T cells reduces disease severity and improves wellness in twi mice

**Figure 2.**
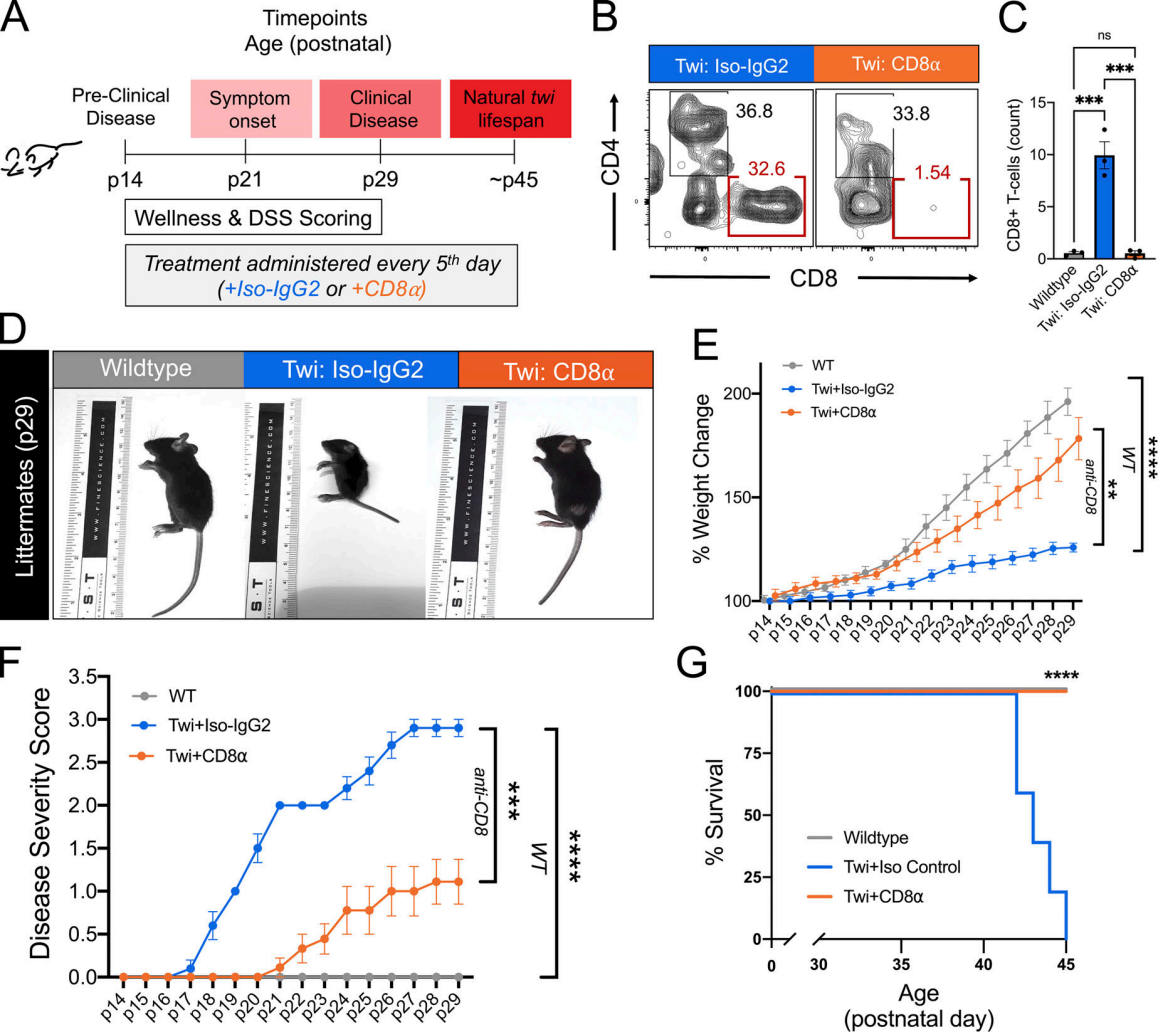
**Depletion of CD8**^**+**^ **T cells decreased clinical disease in twi mice. (A)** Experimental schematic showing the timing of the CD8α or isotype IgG control injections in WT and twi mice. **(B)** Flow plots of brain CD3^+^ T cells (as gated in Fig. S1) in WT, twi*:Iso-IgG2*, twi*:CD8*α at p29 show depletion of CD8^+^ T cells in twi*:CD8α*. **(C)** Administration of CD8α antibody shows specific depletion of CD8^+^ T cells without impacting CD4^+^ T cells; *n* = 3 biological replicates, two independent experiments. **(D)** Representative mice from each treatment group (WT, twi*:Iso-IgG2*, twi:*CD8α*) at p29. **(E)** Percent weight change normalized to p14 showing the weight gain of each treatment group between p14–p29; *n* = 8–10 biological replicates, four independent experiments. **(F)** DSS for each treatment group between p14–p29; *n* = 8–10 biological replicates, four independent experiments. **(G)** Treatment with CD8α extended lifespan of twi mice past the normal twi lifespan; *n* = 5 biological replicates, two independent experiments. **P < 0.01, ***P < 0.001, ****P < 0.0001; statistical tests used were *t* tests (C), two-way ANOVA (E–G).

**Figure S3.**
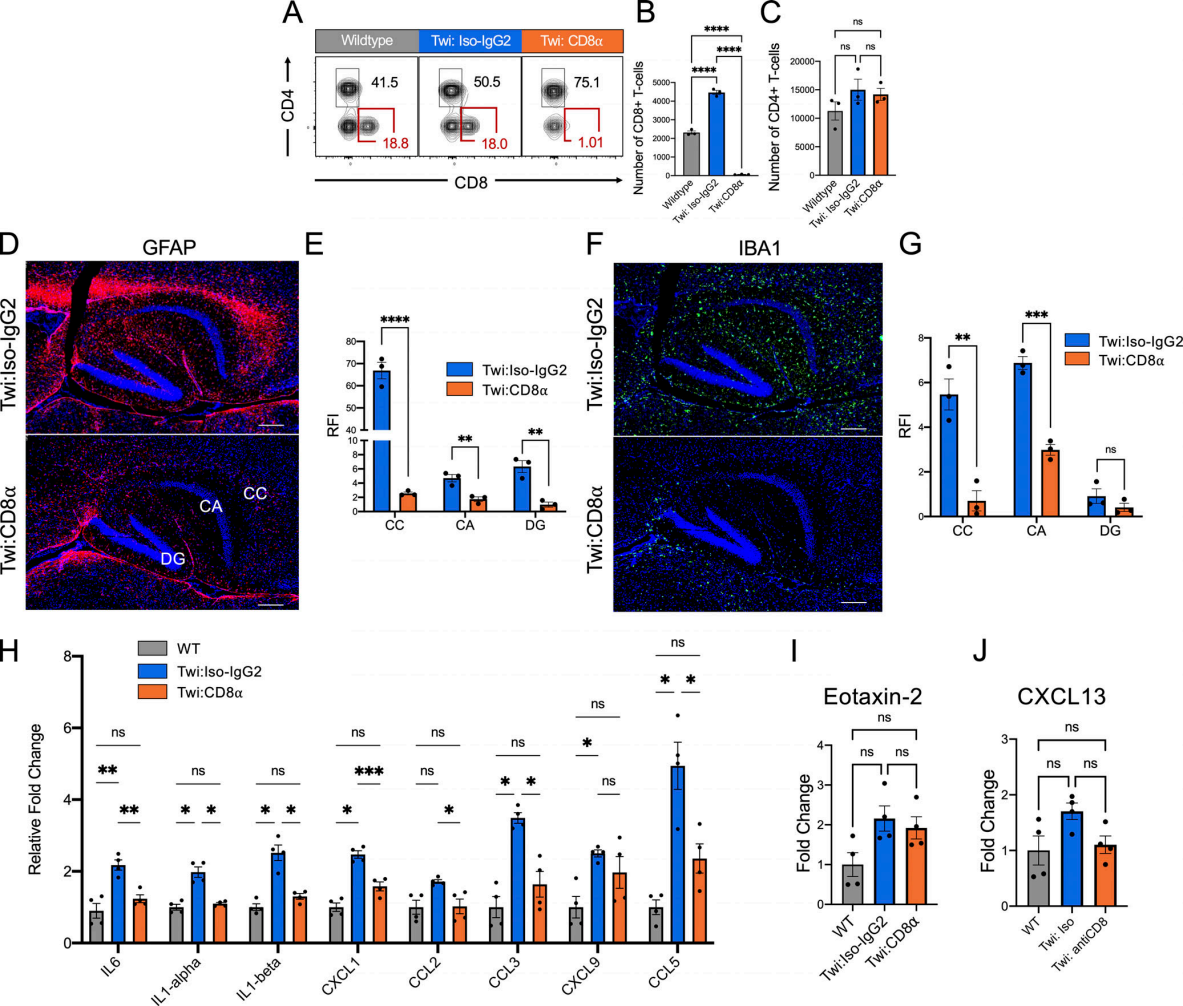
**Activated glia and inflammation are reduced in the twi mice following CD8**^**+**^ **T cell depletion. (A–C)** Representative flow plots of CD3^+^ T cells for WT, twi*:Iso-IgG2*, and twi*:CD8α* spleens at p29 show depletion of CD8^+^ T cells. CD8^+^ T cell depletion was specific to CD8^+^ T cells (B) without impacting the CD4^+^ T-cell population (C); *n* = 3 biological replicates, two independent experiments. **(D and E)** Activated astrocytes (GFAP) are decreased in twi corpus callosum (CC), cornu ammonis (CA), and dentate gyrus (DG) following CD8^+^ T cell depletion; *n* = 5 images/animal averaged, *n* = 3 animals, scale bars are 300 μm. RFI, relative fluorescence intensity. **(F and G)** Activated microglia/macrophages (IBA1) are decreased in twi CC, CA, and DG following CD8^+^ T cell depletion; *n* = 5 images/animal averaged, *n* = 3 animals. Scale bar = 180 μm. **(H)** Fold change of additional cytokines in twi CNS show a decrease following CD8^+^ T cell depletion in twi mice (normalized to WT); *n* = 4 biological replicates, two independent experiments. **(I and J)** Immunoblot analysis of Eotaxin-2 (I) and CXCL13 (J) cytokines are unchanged between treatment groups. *P < 0.05, **P < 0.01, ***P < 0.001, ****P < 0.0001; statistical tests used include one-way ANOVA (B, C, I, and J) and two-way ANOVA (E, G, and H).

To determine the functional importance of CD8^+^ T cells to GLD-like disease in the twi mouse, CD8^+^ T cells were depleted by administration of anti-mouse CD8α antibody (twi:CD8α; 300 µg, i.p.; Willis et al., 2019) every fifth day starting at p14 (Fig. 2, A–C). IgG2b isotype-matched antibody (twi:Iso-IgG2; 300 µg, i.p.) administration did not affect CD8^+^ T cell counts nor did CD8α antibody treatment affect CD4^+^ T cell populations in twi mice (Fig. 2 C and Fig. S3, A–C). Depletion of CD8^+^ T cells in twi mice significantly improved overall wellness (Fig. 2, D and E), and delayed onset and overall severity of disease (Fig. 2, D and F). Reduced disease severity was evident as 10/10 twi:Iso-IgG2 mice developed paralysis; yet, among twi:CD8α mice only 7/9 developed a mild twitching phenotype by p29 as measured by the disease severity scoring (DSS; Marshall et al., 2018; Fig. 2, E and F). Moreover, when compared with the natural twi lifespan of p45, which was used as the experimental endpoint of this study, twi:CD8α mice exhibited increased survival (Fig. 2 G).

### CD8^+^ T cell depletion ameliorates demyelination and reduces neuroinflammation in twi mice

**Figure 3.**
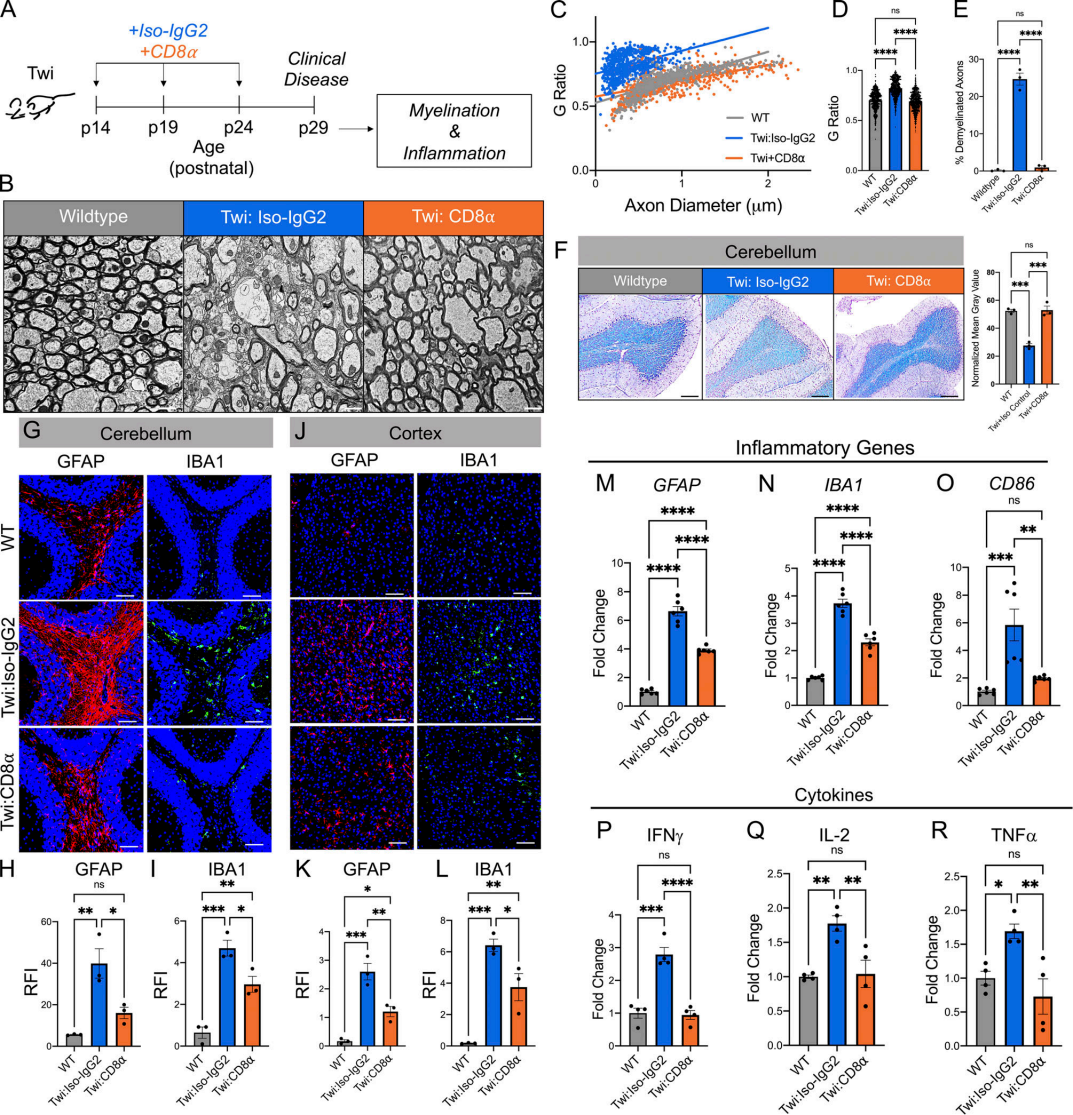
**Depletion of CD8**^**+**^ **T cells ameliorated myelin pathology and attenuated CNS inflammation in twi mice at p29. (A)** Experimental schematic showing treatment regime and analysis timeline. **(B)** Representative images of transmission electron microscopy images of the corpus callosum at p29; scale bars are 1 μm. **(C)** Plotted g-ratio vs. axon diameter at p29; *n* = 250 axons/animal, 3 animals/group. **(D)** G-ratios for all treatment groups at p29; *n* = 250 axons/animal, 3 animals/group. **(E)** Percentage of demyelinated axons (g-ratio >0.95) present in each treatment group at p29, *n* = 3. **(F)** Representative images of Luxol Fast Blue staining of the cerebellum at p29 with calculated normalized mean gray value for image; *n* = 5 images/animal (averaged), *n* = 3 animals/group, scale bars are 100 μm. **(G–I)** GFAP and IBA1 staining in the cerebellum of WT, twi*:IsoIgG2*, and twi*:CD8α* with quantification; *n* = 5 images/animal, 3 animals/group, scale bars are all 100 μm. **(J–L)** GFAP and IBA1 staining in the cortex of WT, twi:*IsoIgG2*, and twi*:CD8α* with quantification; *n* = 5 images/animal, 3 animals/group. Scale bar = 100 μm. **(M–O)** Normalized fold change of gene expression of inflammatory markers known to be upregulated in twi mice are reduced with CD8^+^ T cell depletion; *n* = 6 biological replicates, two independent experiments. **(P–R)** Normalized fold change (to WT) of cytokines from hemibrains of animals from each treatment group; *n* = 4 biological replicates, two independent experiments. *P < 0.05, **P < 0.01, ***P < 0.001, ****P < 0.0001; statistical tests used were *t* tests (D–R).

The impact of CD8^+^ T cell depletion on CNS demyelination and neuroinflammation was also analyzed (Fig. 3 A). Measurement of myelinated axon g-ratios in the corpus callosum of WT, twi:Iso-IgG2, and twi:CD8α mice determined that depletion of CD8^+^ T cells ameliorated demyelination (Fig. 3, B–D) and profoundly decreased the percentage of demyelinated axons from 24.6% in twi:Iso-IgG2 to 0.9% in twi:CD8α mice (Fig. 3 E). These findings were consistent with the preservation of myelin throughout the cerebellum in twi:CD8α mice, as indicated by histological staining of the cerebellum with luxol fast blue (Fig. 3 F). Neuroinflammation was evaluated by IHC staining to examine activated astrocytes (GFAP) and microglia/macrophages (Iba-1). Depletion of CD8^+^ T cells in twi mice showed reduced activated astrocytes and microglia/macrophages in the cerebellum (Fig. 3, G–I), cortex (Fig. 3, J–L), hippocampus, and corpus callosum (Fig. S3, D–G), as compared with twi:Iso-IgG2. These findings were corroborated with RT-qPCR on hemibrains that showed astrocytes (*GFAP*) and microglia/macrophages (*IBA1*, *CD86*) exhibited reduced activation in twi:CD8α mice compared with twi:Iso-IgG2, although these markers still remained elevated when compared with WT littermates (Fig. 3, M–O). Multiplex cytokine arrays also identified significantly decreased levels of IFNγ, IL-2, TNFα, (Fig. 3, P–R), and IL-6, IL-1α, CXCL1, CCL2, CCL3, CXCL9, and CCL5 in twi:CD8α mice compared with twi:Iso-IgG2 mice (Fig. S3 H). Changes in these factors were related to CD8^−^ associated pathology in twi mice, as the expression of other chemokines, such as Eotaxin-2 and CXCL13, did not differ between WT, twi:Iso-IgG2 mice, and twi:CD8α mice (Fig. S3, I and J).

### Single-cell RNA sequencing (scRNAseq) reveals highly activated CD8^+^ T cells with transcriptomic changes in twi mice

**Figure 4.**
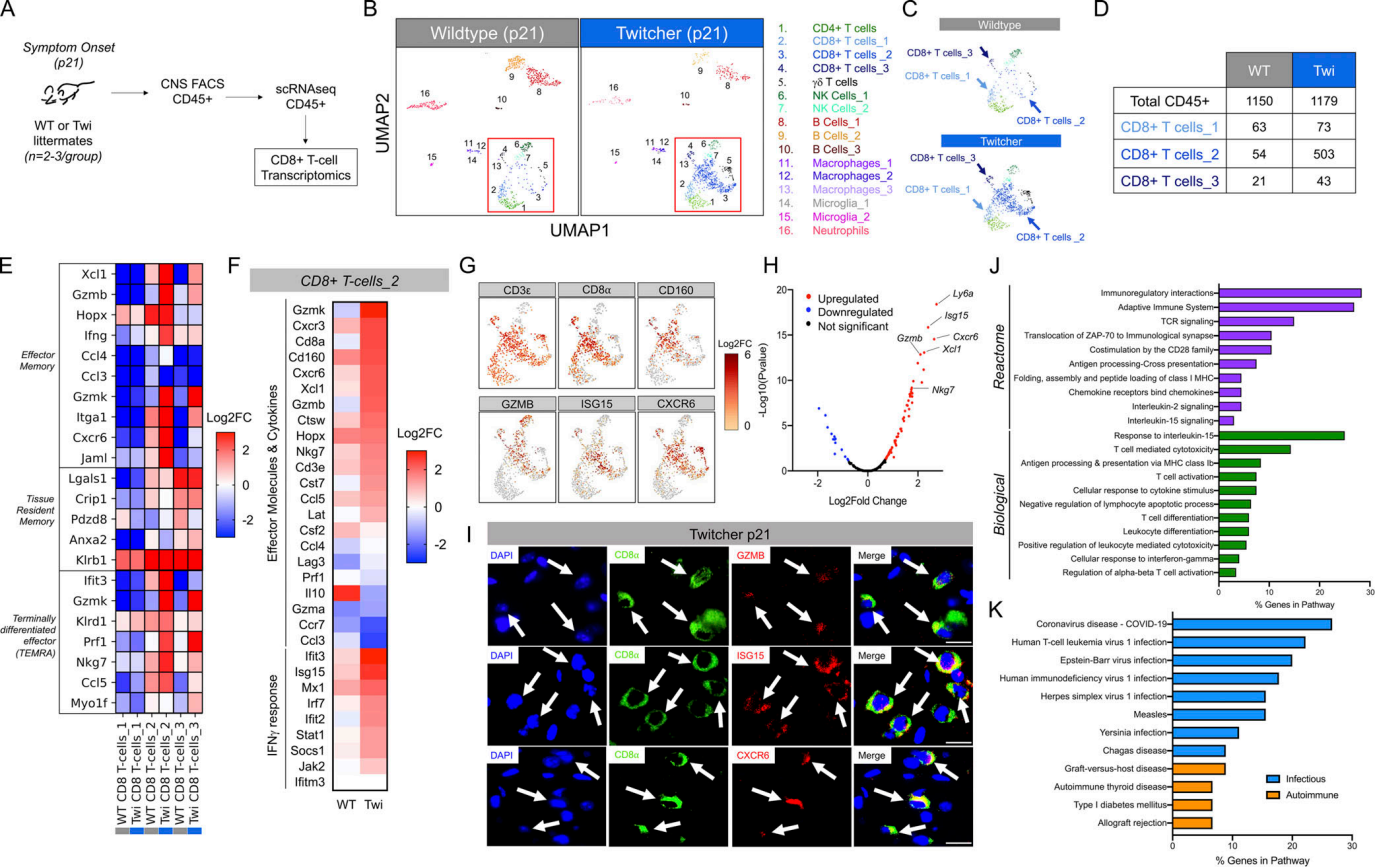
**Transcriptomic analysis of CD8**^**+**^ **T cells in twi CNS at disease onset (p21). (A)** Experimental workflow for isolating CNS CD45^+^ cells for analysis by scRNAseq; *n* = 2–3 animals/group. **(B)** UMAP visualization of scRNAseq data of CD45^+^ populations from p21 WT and twi mice shows 16 unique populations. **(C)** The twi CD8+T cells_2 subpopulation showed an expansion compared to the WT CD8+T cells_2 population. **(D)** Raw counts for each CD8^+^ T cell population identified for WT and Twi by scRNAseq. **(E)** Heatmap showing further characterization of three identified CD8^+^ T cell populations. **(F)** Effector molecules, cytokines, and genes involved in the IFNγ response showed increased expression in this twi CD8^+^ T cell population compared with WT. **(G)** UMAP plots showing cytotoxic genes are highly expressed by this twi CD8+T subpopulation. **(H)** Volcano plot showing DEGs in twi CD8^+^ T cell subpopulation vs. WT (upregulated = red; downregulated = blue). **(I)** Immunohistochemical validation of three significantly upregulated genes in CD8^+^ T cells in twi brains; scale bars are 20 μm. **(J)** Significant GO analysis results for biological and reactome processes analyzing the pathways associated with the 67 upregulated genes. **(K)** Significant KEGG pathway analysis results for the 67 upregulated genes in the twi CD8^+^ T cell subpopulation defined as CTLs.

To provide a profile of the immunological landscape in the twi mouse brain, we performed scRNAseq of CNS infiltrating CD45^+^ cells at p21 (twi mice compared with WT littermates; Fig. 4, A and B). This analysis identified five populations of T cells including CD4^+^, CD8^+^, and γδ T cells (Fig. 4 B). Of the three CD8^+^ T cell populations identified, one showed a ninefold increase in CD8^+^ T cells in twi mouse brain when compared with WT (Fig. 4, C and D). This observation validated our earlier findings and was also consistent with previous reports which observed CD8^+^ T cells in twi and human GLD CNS (Ohno et al., 1993; Matsushima et al., 1994; Taniike et al., 1997; Wu et al., 2000; Iacono et al., 2022). Transcriptomic analyses of the three CD8^+^ T cell populations in twi mouse brains showed that one of these, denoted as “CD8+ T cells_2” (Fig. 4 C), had elevated expression of effector memory and terminally differentiated effector T cells genes. This population also had increased expression of many effector molecules, particularly cytokines and genes involved in the IFNγ response (Fig. 4, E and F; Szabo et al., 2019). Additionally, this population was confirmed to have an activated cytotoxic T cell (or CTL) phenotype, as indicated by the increased expression of cytotoxic markers including *CD160*, *ISG15*, *GZMB*, and *CXCR6* (Fig. 4 G). A volcano plot showed 81 differentially expressed genes (DEGs) identified in this twi CD8^+^ T cell population, of which 67 were significantly upregulated (Fig. 4 H). We verified several of the notably expressed genes by immunohistochemical staining in p21 twi brains (Fig. 4 I). This analysis identified clusters of CD8^+^ T cells, particularly in the cerebellar cortex, where colocalization of three of the most significantly upregulated genes, *GZMB*, *ISG15*, and *CXCR6*, within CD8^+^ cells was notable (Fig. 4 I). Gene ontology (GO) analyses of the upregulated genes revealed twi CD8^+^ T cell involvement in several biological and reactome pathways including TCR signaling, T cell activation, T cell differentiation, positive regulation of leukocyte-mediated cytotoxicity*,* and several pathways involving antigen presentation such as antigen processing and presentation via MHC class Ib and costimulation by the CD28 family (Fig. 4 J). Furthermore, Kyoto Encyclopedia of Genes and Genom (KEGG) pathway analysis showed that the upregulated genes in this CD8^+^ T cell population are implicated in infectious and autoimmune processes (Fig. 4 K). The top six significant processes identified were processes related to cellular responses to viral infections and indicated comparisons to Coronavirus disease-COVID19, human T cell leukemia virus 1 infection, EBV, HIV1 infection, HSV1 infection, and measles. These data provide a basis for evaluating potential underlying mechanism of CD8^+^ T cell activity in twi mice.

### Enhanced activation of CD8^+^ T cells from brain of twi mice

**Figure 5.**
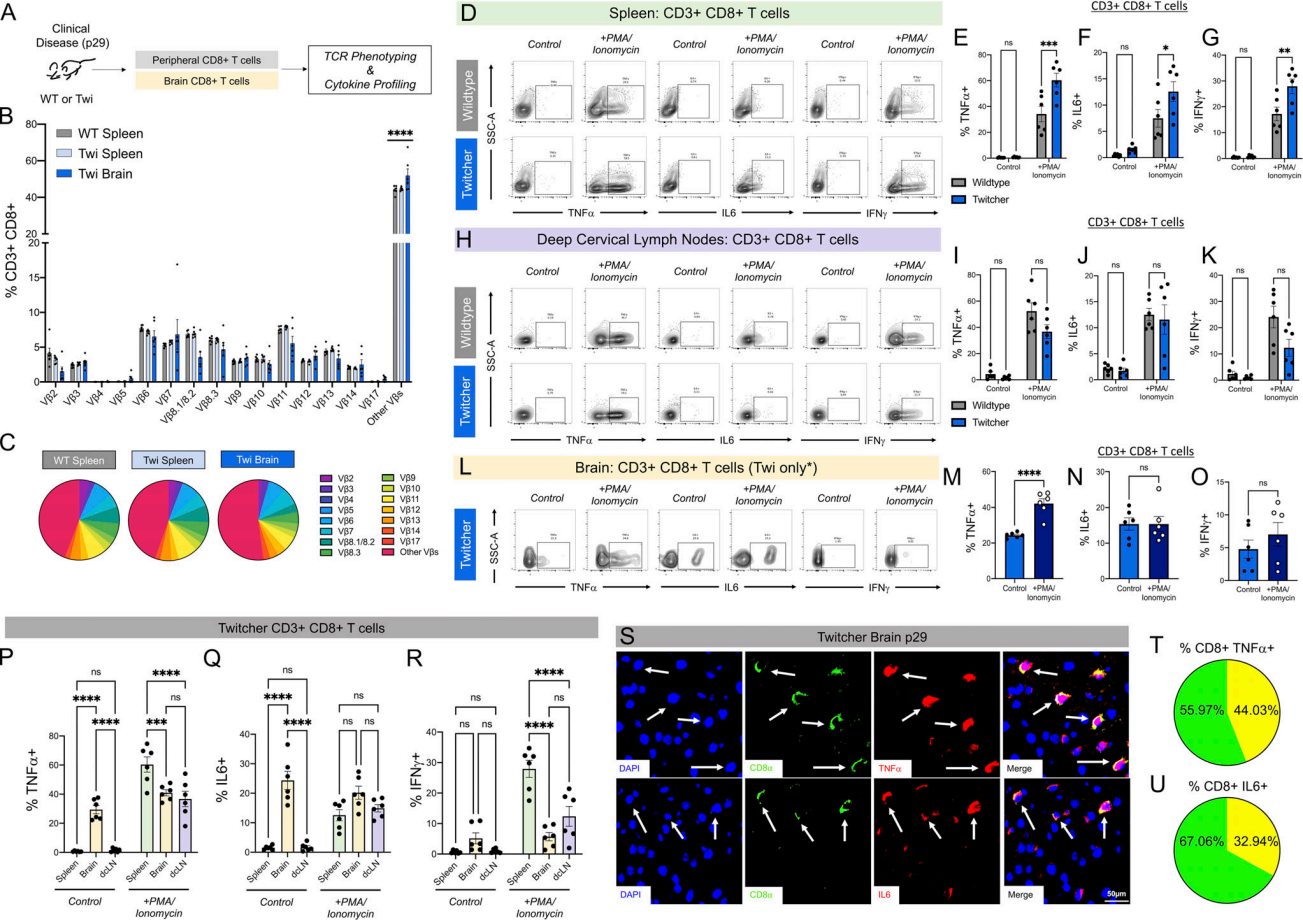
**TCR phenotyping and intracellular cytokine stimulation of CD8**^**+**^ **T cells in WT vs. twi mice at p29. (A)** Experimental schematic showing TCR and cytokine profiling for CD8^+^ T cells was performed on CD8^+^ T cells from brain and peripheral organs at p29. **(B)** TCR profiling of 15 Vβs on CD8^+^ T cells isolated from WT spleen, twi spleen, and twi brain. **(C)** Pie charts showing the percentages of CD8^+^ T cells with each Vβ. **(D–G)** Representative flow cytometry plots showing splenic WT vs. twi CD8^+^ T cell responses to stimulation with PMA/ionomycin. Percentages of CD8^+^ T cells expressing TNFα (Ε), IL6 (F), and IFNγ (G) were all increased in twi* +* PMA/ionomycin stimulated compared with WT, but did not differ between groups at baseline. **(H)** Representative flow cytometry plots showing WT vs. twi CD8^+^ T cell responses to stimulation with PMA/ionomycin in dcLNs. **(I–K)** Percentages of CD8^+^ T cells expressing TNFα (Ι), IL6 (J), and IFNγ (K) showed no differences in baseline or response to PMA/ionomycin stimulation between WT and twi. **(L–R)** Representative flow cytometry plots showing twi CD8^+^ T cell responses to stimulation with PMA/ionomycin in brain. Percentages of twi CD8^+^ T cells expressing TNFα (Μ) increase following stimulation, but IL6 (N) and IFNγ (O) do not change. Evaluation of twi CD8^+^ T cell cytokine production between spleen, brain, and dcLNs show that twi brain CD8^+^ T cells constitutively express TNFα (P) and IL6 (Q) but not IFNγ (R) without PMA/ionomycin stimulation, which is not present in twi spleen or dcLNs. **(S)** Immunohistochemical validation showing TNFα and IL6 colocalization with CD8^+^ T cells in twi brains at p29; arrows denote CD8^+^ cells that co-labeled with cytokine; scale bars are 50 μm. **(T and U)** Pie charts showing the percentage of CD8^+^ TNFα^+^ (T) and CD8^+^ IL6^+^ (U) T cells out of total CD8^+^ T cells in p29 Twi brains. Each pie chart percentage was calculated by averaging counts from three biological replicates. *P < 0.05, **P < 0.01, ***P < 0.001, ****P < 0.0001; statistical tests used were *t* tests (M–O) and two-way ANOVAs (E–G, I–K, and P–R).

To gain an understanding of an underlying pathogenic mechanism by which CD8^+^ T cells in twi brain could contribute to pathology, we determined whether CD8^+^ T cells from twi mice exhibited clonal expansion (clonality) by analyzing the TCR repertoire (Fig. 5 A). We quantified the expression of 15 of the most common TCR Vβs on CD8^+^ T cells from both brain and spleen by flow cytometry and compared twi with WT littermates (Fig. 5 B). Our results did not show obvious expansion of any specific TCR Vβs on CD8^+^ T cells in twi mice when isolated from either brain or spleen (Fig. 5 B). Interestingly, a possible difference in the percent of CD8^+^ T cells in the “Other Vβs” category was noted in twi brains compared with twi spleens or WT spleens (Fig. 5, B and C). This category included all CD8^+^ T cells that were not represented by any of the other specifically tested Vβs. However, due to the abundance of CD8^+^ T cells in the twi brain and scarcity of CD8^+^ T cells in naive WT brains, a direct and valid comparison of TCR Vβs on CD8s from twi brain with WT brain was neither feasible nor reliable.

We next investigated how CD8^+^ T cells from spleens, dcLNs, or brains from WT and twi littermates differed in terms of stimulated cytokine production using an ex vivo intracellular cytokine staining assay. Cells were cultured in Brefeldin A either with or without PMA/ionomycin stimulation for 4–5 h and then the production of TNFα, IL6, and IFNγ was measured using flow cytometry. Baseline (unstimulated) cytokine production from spleens of WT and twi mice did not differ; however, when stimulated from twi it showed enhanced production of TNFα, IL6, and IFNγ (Fig. 5, D–G). In contrast, CD8^+^ T cells isolated from the dcLNs of WT and twi exhibited similar baseline levels and comparable cytokine responsiveness when stimulated (Fig. 5, H–K). Interestingly, CD8^+^ T cells isolated from the CNS of twi brains also exhibited elevated production of TNFα, but not IL-6 or IFNγ, following stimulation (Fig. 5, L–O). When these data for CD8^+^ T cells from twi were analyzed across the various organs, we found higher baseline cytokine production of either TNFα or IL-6, but not IFNγ, from the CD8^+^ T cells isolated from the twi brain, even without PMA/ionomycin stimulation (Fig. 5, P–R). This difference in stimulated cytokine production was not seen in CD8^+^ T cells isolated from twi spleens or dcLNs (Fig. 5, P–R). IHC for TNFα and IL-6 on twi (p29) brain tissue samples also validated that CD8^+^ T cells in twi brains constitutively expressed these cytokines (Fig. 5 S).

Herein, we have defined a novel role for CD8^+^ T cells in the initial development of GLD disease and myelin pathology. We have identified that there is spontaneous development of a robust, pathological CD8^+^ T cell population that is associated with GLD disease in this naturally occurring disease model. It is currently not known how the metabolic consequences of *galc* mutations in GLD result in the development of such a profound immunological response. CD8^+^ T cells are a predominant cytotoxic lymphocyte population found in human neuroinflammatory diseases affecting CNS white matter, including GLD (Iacono et al., 2022; Gate et al., 2020; Wagner et al., 2020; Evans et al., 2019). Accordingly, additional study on the genesis of CD8^+^ T cell responses in this disease model may contribute to our fundamental understanding of how these immunological responses may begin. It is important to point out that this model has some striking parallels and differences from other known autoimmune disease models. First, CD8^+^ T cell responses in twi mice were spontaneous and occurred very early in postnatal development. It is important to note that immune cell irregularities have been reported in other lysosomal storage diseases, which suggest that findings herein may have implications for immune-mediated pathology in similar lysosomal storage diseases, but to date, a role for CD8^+^ T cells has not been tested directly in other leukodystrophies. Future studies on the origins and differentiation of these CD8^+^ T cell populations in twi mice may hold particular importance for both their similarities and differences with CD8^+^ T cells in other leukodystrophies and experimental neuroimmune models.

### Characterization of CD8^+^ T cells in twi mice

Toward the goal of characterizing the CD8^+^ T cell populations in this disease model, our use of TCR phenotyping, scRNAseq, and profiling of cytokines have generated salient findings. First, TCR profiling of Vβ on CD8^+^ T cells did not identify an obvious clonal expansion of the populations analyzed. It is important to qualify that the approach used was not exhaustive and we did note that differences within a yet-to-be-defined Vβ subpopulation may yield a unique clonal population. Our scRNAseq analyses of gene expression within CD45^+^ population in twi brains also determined that there were no significant differences in expression of CD1d, an obligate cofactor for TCR-mediated activation to lipids, which would suggest that the lipid psychosine may not itself be an antigen in any proposed autoimmune mechanism in this disease model. Next, transcriptomics also defined the prominent CD8^+^ T cell population in the twi brain as expressing a cytotoxic phenotype. Consistent with this, our results also demonstrated a spatial colocalization of cytotoxic markers, such as *GZMB*, in CD8^+^ T cells within the twi CNS. When considered with the profound protection of CNS myelination upon depletion of CD8^+^ T cells in the twi mice, these data support a deleterious role for CD8^+^ T cells in this disease model. At present, it is unclear whether CD8^+^ T cells are antigen-driven, potentially directly acting upon oligodendrocytes to evoke demyelination, or if the heightened production of cytokines, such as TNFα and IL-6, from CD8^+^ T cells are meditated in the loss of myelin in this disease. Future studies that may adopt transcriptomic approaches to explore TCR spectratyping with greater depth may define a clonally expanded population in twi or GLD, while additional genetic approaches may elucidate specific functions of CD8^+^ T cells necessary for their pathogenic actions in this disease setting.

In this twitcher mouse model, the development of a robust CD8^+^ T cell response was spontaneous. This naturally occurring CD8^+^ T cell–mediated phenotype is similar to some other CNS models (Mohebiany et al., 2020; Gross et al., 2019) but also distinguishes it from most other widely adopted models of CNS autoimmune demyelination, which typically involve inoculation with peptide, infection with a virus, or activation of a transgene to evoke measurable antigen-specific responses, and have been used to great benefit for the study of multiple sclerosis (MS) or neuromyelitis optica (Steinman and Zamvil, 2006). Interestingly, our KEGG analysis of the DEGs in twi CD8^+^ T cells when compared with other CNS diseases identified pathways associated with infectious and autoimmune processes. Of note, this analysis indicated a striking similarity in the transcriptional responses of CD8^+^ T cells in the twi CNS to responses observed in viral infections. From this observation, and when we consider that there was no obvious clonal expansion of a TCR-defined CD8^+^ T cell population in twi mice, these data may suggest a “cytokine storm” as one possible mechanism of CD8^+^ T cell–mediated neuropathology, which is known in other settings to result in an intense and tissue destructive process. While at this time we cannot pinpoint any one specific mechanism of CD8^+^ T cell–mediated disease, there also remain other important and compelling questions about the roles of CD8^+^ T cells in this disease. For instance, what factor(s) initiate the CD8^+^ T cell–mediated responses in twi? What cues are driving the specific recruitment of the CD8^+^ T cells to, and activation within, the CNS? How does innate immunity contribute to this process? While the answers to these questions are currently unclear, study of these questions and the CD8^+^ responses in twi may have potential relevance to a range of this and other neurological diseases.

It was also noteworthy that the development of CD8^+^ T cell responses directed at the CNS in this twi mouse model also differ from most other CNS demyelinating disease models because there appears to be rapid accumulation of highly activated CD8^+^ T cells within the CNS that was not preceded by vigorous changes in CD8^+^ T cells in peripheral organs. In fact, we observed significant differences in activated CD8^+^ T cells within the CNS compartment of twi mice within 2 wk of birth while the peripheral CD8^+^ responses were unremarkable by comparison. This observation may suggest a fundamental difference in both where cells become highly activated and how they are stimulated. First, the activation of CD8^+^ T cells within the CNS compartment may indicate a natural difference in the process of neuroinflammation in this disease where the CD8^+^ T cells engage APCs as they enter the CNS or perhaps even within the CNS itself. This observation would also potentially implicate either CNS macrophages, which are themselves robustly activated in GLD, or atypical APCs within the CNS itself, such as glial cells (Kirby et al., 2019), as the more likely means of local activation in this disease setting. As mentioned, we do not presently know the antigens or clonality of the CD8^+^ T cell populations in this model or the human disease condition. The absence of CD1d changes in the T cell populations by scRNAseq would suggest that a lipid, such as psychosine itself, is not likely the antigen driving these responses. Nevertheless, we have provided evidence that the robust differences in CD8^+^ T cell populations are noted across species, including mouse, canine, and human disease settings. Hence, the findings of this study potentially provide important information on a novel disease-relevant process.

### Concluding remarks

An important implication of our findings is that these data portend a potential therapeutic benefit for the treatment of GLD by therapeutics that target T cells. For example, some disease-modifying treatments for MS have established applications for treating pediatric patients that could then have applications for GLD as well (Krysko et al., 2018; Béchet et al., 2020). While we have demonstrated benefit to GLD disease in twi mice by affecting CD8^+^ T cells, the long-term impact of this approach is yet to be determined. Nevertheless, the effect of this approach on early GLD neuropathology may represent a means by which to enhance existing therapies. For instance, the current therapy for GLD, hematopoietic stem cell transplantation (Escolar et al., 2005), is limited by a very narrow window for treatment, often within the first month of life (Escolar et al., 2016). Since newborn screening for GLD is not widely available (Wasserstein et al., 2021; Schrier Vergano et al., 2022), new therapeutic approaches such as immunotherapies may be warranted. Applying immunotherapies could extend the therapeutic window for hematopoietic stem cell transplantation (Kvistad et al., 2022) or enhance long-term outcomes from interventions such as gene therapy (Heller et al., 2021; Hordeaux et al., 2022), and therein provide greater benefit to more patients. Future studies to address these issues should examine whether existing FDA-approved immunomodulatory therapeutics can modify GLD disease, both as a monotherapy and as a means to mitigate the early stages of disease and potentially expand eligibility among patients to qualify for other therapies.

In conclusion, our findings increase the fundamental understanding of how GLD pathology occurs and suggest salient insights that may support novel therapeutic approaches to treat GLD. Furthermore, these data have potential relevance for other neurological diseases in which CD8^+^ T cells have been implicated, yet their origins remain enigmatic, such as Alzheimer’s disease (Altendorfer et al., 2022; Gate et al., 2020), Parkinson’s disease (Williams-Gray et al., 2018; He et al., 2022), or amyotrophic lateral sclerosis (Campisi et al., 2022; Rentzos et al., 2012). Hence, we propose that the development of CD8^+^ T cells in this model represents a unique opportunity to study the natural development of CD8^+^ T cell responses that appear to target the CNS.

## Materials and methods

### Animals

All procedures involving animals were conducted with approval from the Institutional Animal Care and Use Committee at the University of Connecticut School of Medicine in accordance with guidelines set forth by the National Research Council of the National Academies Guide for the Care and Use of Laboratory Animals. Mice used in this study were WT C57BL/6J (strain #000663) and twitcher C57BL/6J (strain #000845). Mice used in this study were litter-matched pups with confirmed genotypes using the following primers (Forward: 5′-GCT​TGG​AAT​TTG​GTG​GCT​AG-3′; Reverse: 5′-GAT​GGT​GAG​GTT​TCC​CAA​GC-3′; Weinstock et al., 2020). Dog tissue used in this study was kindly received from co-author Dr. Allison Bradbury (Bradbury et al., 2016; Corado et al., 2020).

### Human brain tissues

Deidentified samples from GLD and age-matched control patient (1–2 yr old) paraffin-embedded brain tissue were obtained from the National Institute of Child Health and Human Development Brain and Tissue Bank for Developmental Disorders.

### Brain single-cell isolation

At the desired time point, animals were euthanized using isoflurane and then received cardiac perfusion with 1X PBS. Hemibrains at each time point were collected and their meninges removed. Hemibrains were then enzymatically dissociated using collagenase D (12.5 mg/ml) and DNase I (1 mg/ml) for 30 min at 37°C. CNS tissue was resuspended into 10 ml HBSS+ containing 10% FBS to neutralize enzymatic activity. Tissue was then strained with a 70-µm cell strainer and centrifuged at 1,000 RPM for 5 min at 4°C. The pellet was resuspended in 30% Percoll, carefully underlaid with 70% Percoll, then spun at 300 × *g* for 23 min at 4°C. Myelin debris was removed from the top layer and leukocytes were collected from the interface between the 30 and 70% Percoll layers. Collected leukocytes were resuspended in 10% FBS in HBSS+ and centrifuged at 1,000 RPM for 5 min at 4°C. Samples were either prepared for the intracellular cytokine assay or were stained and analyzed as described below in the Flow cytometry methods section.

### Spleen single-cell isolation

At the desired time point, animals were euthanized using isoflurane and then received cardiac perfusion with 1X PBS. Spleens were removed and crushed through a 70-µm cell strainer, washed with 1X PBS, then centrifuged at 1,000 RPM for 4 min. Samples were resuspended in 5 ml of Red Blood Cell Lysis Buffer for 5 min at RT, washed with 45 ml of 1X PBS, and centrifuged at 1,000 RPM for 4 min. Cells were counted and 1 × 10^5^ cells were isolated from each spleen for staining. Samples were either prepared for the intracellular cytokine assay or stained and analyzed as described below in the Flow cytometry methods section.

### dcLN single-cell isolation

At the desired time point, animals were euthanized using isoflurane and then received cardiac perfusion with 1X PBS. dcLNs were removed as described (Maloveska et al., 2018) and crushed through a 70-µm cell strainer, washed with 1X PBS, then centrifuged at 1,000 RPM for 4 min. Cells were counted and 1 × 10^5^ cells were isolated from each sample for staining. Samples were either prepared for the intracellular cytokine assay or stained and analyzed as described below in the Flow cytometry methods section.

### Intracellular cytokine assay

Cells were plated in complete tumor medium (MEM, dextrose, essential amino acids, non-essential amino acids, sodium pyruvate, sodium bicarbonate, gentamycin, penicillin g, streptomycin sulfate, 2-mercaptoethanol). Cells were treated with Brefeldin A (BD Biosciences) and stimulated with PMA/ionomycin (Invitrogen) for 4–5 h at 37°C. Following incubation, cells were washed with 1X PBS, centrifuged at 1,000 RPM for 3 min, then stained with extracellular antibodies for 20 min on ice. Samples were then washed twice with 1X PBS and fixed with 1.5% paraformaldehyde (PFA) for 5 min on ice followed by 10 min at room temperature. Samples were next washed with 2% Saponin in HBSS, centrifuged, and permeabilized with 2% Saponin for 15 min on ice. Intracellular antibodies were added to samples and incubated at 4°C overnight. The next morning, samples were washed twice with 1X PBS, resuspended in 200 μl of 1X PBS, and analyzed with LSR II (Becton Dickinson).

### Flow cytometry

Samples for flow cytometry analysis were stained with primary conjugated antibodies (Table S1) for 20 min at 4°C. Samples were then resuspended in 1 ml of 1X PBS, centrifuged, and resuspended in the 200 μl of 1X PBS for flow analysis on LSR II (Becton Dickinson). Single color compensation controls and fluorescence minus one controls were prepared for each experiment to account for differences in detector sensitivity across experimental time points. Fig. S1 shows the gating strategy for identification of T cell populations in brain, spleen, and dcLN.

### Histology and IHC

At p29, mice were perfused with 10 ml of 4% PFA and then hemibrains were left to fix in 4% PFA for 24 h. Tissues were embedded in paraffin and then sliced into 8-µM sections and placed on superfrost plus glass slides. All sections (mouse, dog, and human) were heated for 30 min at 60°C, then rehydrated using three washes of 100% xylene for 5 min each, followed by 5-min washes in a decreasing ETOH gradient (100, 95, 70%). Slides were then washed with 1X PBS and deionized H_2O_ (diH_2_O) and stained either with luxol fast blue (LFB) or prepared for IHC by performing antigen unmasking in 0.01 M citrate acid buffer. Slides for IHC were blocked for 1 h at room temperature in blocking buffer (0.1% Tween, 10% normal goat serum, in 1X PBS), then incubated in primary antibodies (in blocking buffer) overnight at 4°C (see Table S1 for antibody details). Following overnight incubation, slides were washed five times for 5 min each with 1X PBS and incubated for 1 h at room temperature with the appropriate secondary antibody (in blocking buffer). Slides were washed five times with 1X PBS, counterstained with DAPI for 5 min, then washed and mounted. Sections stained with LFB were imaged at 10× with Olympus IX71 and processed using ImageJ software (National Institutes of Health). Each image was converted to grayscale, then the mean gray value was measured and normalized to the background of each image. IHC-labeled sections were imaged with Leica Thunder Microscope and relative fluorescence intensity was processed using Image J software (National Institutes of Health).

### In vivo injections and disease severity scoring

Twitcher mice received either anti-CD8α antibody (Twi:CD8α; 300 µg, i.p.) or isotype-matched control antibody (Twi:Iso-IgG2; 300 µg, i.p.) every fifth day starting at p14. Mice underwent daily clinical scoring which included weight measurements and evaluation of tremor and locomotion. Weight loss, tremor, and locomotion severity were measured daily and used to calculate the DSS, an established method for measuring Twitcher disease progression (Santambrogio et al., 2012; Marshall et al., 2018). Total body weight loss received a score from 0 to 4, with 0 being a loss of 0–0.25 g/d and 4 being a loss of over 1.0 g/d. Locomotion was also scored from 0 to 4, with 0 being normal gait, 2 being evidence of one impaired hindlimb, and 4 being moribund. Tremor was binary, with 0 being no tremor present and 1 indicating the presence of a tremor.

### Transmission electron microscopy

At p29, mice were perfused with 10 ml of fixative containing 2% PFA and 2.5% glutaraldehyde in 0.1 M cacodylate buffer (in diH_2_O). The entire brain was isolated and then incubated in fixative for 1 h at room temperature. After fixing, a 1 mm slice of corpus callosum from each sample was isolated, washed with 0.1 M cacodylate buffer, and then further fixed in 1% OsO4, 0.8% ferricyanide in 0.1 M cacodylate buffer at room temperature for 1 h. Samples were washed five times with diH2O, then underwent en block staining in 1% uranyl acetate (in diH_2O_) at room temperature for 1 h. Samples were then dehydrated using 10-min washes of an increasing ETOH gradient (50% ETOH, 75% ETOH, 95% ETOH) at room temperature and then washed with 3–10 min washes of 100% ETOH at room temperature. Samples were next washed with propylene oxide (PO) twice for 5 min each and then underwent resin infiltration using PolyBed812 with DMP-30 2, 4, 6-Tris (dimethylaminomethyl) phenol (DMP-30) mixed with differing ratios of PO:Resin (first 1:1 for 1 h at room temperature, then 1:3 overnight at room temperature). Finally, samples were incubated for 24 h in 100% resin, then embedded and polymerized at 60°C for 48 h. Each sample was cut into ultrathin 70-nm sections, placed on grides, and postfixed in PO. Samples were visualized using the Hitachi H7650 transmission electron microscope, and all g-ratio and axon diameter measurements were calculated using ImageJ Software (National Institutes of Health).

### Quantitative real-time PCR (qRT-PCR)

Total RNA was isolated from saline-perfused unfixed hemibrains from p29 mice using TRI Reagent (Sigma-Aldrich) that was added to each sample according to the manufacturer’s protocol. cDNA was amplified from isolated RNA via reverse transcription (iScript cDNA synthesis kit, BioRad) and qPCR was performed using specific validated primer pairs for *GFAP*, *IBA1*, and *CD86* (Table S2; Integrated DNA Technologies) and SsoAdvanced Universal SYBR Green Supermix (BioRad). CFX Connect Real-Time PCR Detection System (BioRad) was used to analyze the amplified cDNA. Primers for β-actin were used as the housekeeping gene to normalize gene expression among samples. The relative expression of target RNA was calculated using the comparative cycle threshold analysis (ΔΔCT).

### CNS tissue isolation for cytokine array

Mice were perfused with 10 ml of ice-cold PBS and whole brain was extracted. Hemibrains were added to glass beads and RIPA Lysis buffer with protease inhibitors and then underwent homogenization for 60 s. The homogenate was collected, then centrifuged at 12,000 × *g* for 10 min at 4°C to remove insoluble material. The protein concentration of each sample was determined using a bicinchoninic acid assay and then normalized. The cytokines were analyzed using a membrane-based cytokine array (Mouse Inflammation Antibody Array C1; Raybiotech) that analyzes 40 inflammatory cytokines. All materials used were from this kit. Briefly, the membranes were blocked with blocking buffer and then 1 ml of each sample was added to each membrane and left to incubate overnight at 4°C. The membranes were then washed 3× each with the wash buffers provided, followed by a 2-h incubation with biotinylated antibody cocktail at room temperature. The washes were repeated and then the membranes were incubated with HRP-Streptavidin for 2 h at room temperature. Following another wash set, membranes were incubated with detection buffer for 2 min, then imaged using chemiluminescence. Data were extracted using ImageJ to obtain spot signal densities from each image, then analyzed using the analysis workbook provided by Raybiotech.

### scRNAseq

For samples prepared for scRNAseq, each sample was stained with primary conjugated CD45 antibody and sorted into CD45^+^ and CD45^−^ cell populations. Samples (*n* = 2–3) were pooled for each genotype due to low cell counts. CD45^+^ flow cytometry sorted (FACS) cells were sent to Jackson Laboratory for scRNAseq processing. Briefly, cells were loaded into a Chromium v2 Single Cell Chip and barcoded with unique identifiers using lyso-modified oligonucleotides according to the manufacturer’s instructions (10X Genomics). Each sample contained ∼1,400 cells and had around 14,800 genes detected. Quality control was performed on each sample using three filtering passes. During FASTQ generation, reads with more than 1 mismatch in the 8bpi7 index were excluded. Next during alignment (using STAR), only reads with MAPQ scores >255 that were aligned to the annotated transcripts were retained. Finally, reads containing bases with Q30 scores below 3 were excluded. Following alignment, cell bar barcodes were filtered (up to one mismatch) against a whitelist of 737,500 barcodes provided by 10X Genomics. Then barcodes associated with cells were distinguished from those associated with ambient mRNA using an adaptively computed unique molecular identifiers threshold. To avoid dead cells and other artifacts, we also removed all cell-identifying barcodes where >15% of molecules matched genes expressed in the mitochondrial genome (Yuan et al., 2018). Samples were filtered independently and then aggregated together using batch correction software Harmony (Korsunsky et al., 2019). After quality control, the expression profiles of each cell at the 2,000 most highly variable genes as measured by dispersion (Satija et al., 2015; Zheng et al., 2017) were used for neighborhood graph generation and dimensionality reduction with Uniform Manifold Approximation and Projection (UMAP; Becht et al., 2018). The Leiden community detection algorithm was used to perform clustering (Traag et al., 2019), and clusters were annotated using CellMarker (Zhang et al., 2019). Subclustering was performed ad hoc on a per-cluster basis to separate visually distinct subpopulations of cells. These scRNAseq datasets have been made available at the Gene Expression Omnibus (accession #GSE233320).

### GO and KEGG pathway analysis

The DEGs (P < 0.05) identified by scRNAseq between the WT and twi CD8^+^ T cell populations were collected. We analyzed this gene list using the PANTHER Pathway analysis tool (http://pantherdb.org/) linked through the GO Consortium’s online database (http://www.geneontology.org/; Mi and Thomas, 2009; Mi et al., 2021). Gene names were copied into the PANTHER Pathway analysis tool, and organism (*Mus musculus*), specific enrichment analysis (Biological or Reactome), statistical test type, and correction (Fisher’s Exact with calculation of false discovery rate [FDR]) were selected. Significant results were identified by filtering by FDR (FDR < 0.05) and P value (P < 0.01). We also analyzed this gene list using the KEGG pathways tool (https://www.genome.jp/kegg/). Pathways were considered significant when their adjusted P value was <0.05 (adjusted from raw P value using the Benjamini and Hochberg multiple testing correction).

### Statistical analysis

Appropriate statistical analysis was performed for each experiment using GraphPad Prism version 9 for Mac OS X (GraphPad Software). Differences were considered significant when P < 0.05. Data are presented as mean ± SEM.

### Online supplemental material

Fig. S1 shows validation of CD8^+^ T cell infiltration into the CNS of infantile human GLD and the dog model of infantile GLD. Fig. S2 shows that CD4^+^ T cells influx into the twi CNS only at end-stage disease (p40) and that there are no robust changes in CD8^+^ or CD4^+^ populations in the twi spleen during disease progression. Fig. S3 shows validation that the CD8^+^ T cells were also depleted peripherally in twi mice. Fig. S3 also shows that depletion of CD8^+^ T cells results in decreased astrocyte and microglia/macrophage activation in twi hippocampus as well as decreased inflammatory cytokines known to be upregulated in twi mice. Table S1 lists antibodies used in this study. Table S2 lists RT-qPCR primers used in this study.

## Supplementary Material

Table S1 lists antibodies.

Table S2 shows RT-qPCR primers.

## Data Availability

scRNAseq datasets have been made available at the Gene Expression Omnibus (accession #GSE233320).

## References

[bib1] Altendorfer, B., M.S. Unger, R. Poupardin, A. Hoog, D. Asslaber, I.K. Gratz, H. Mrowetz, A. Benedetti, D.M.B. de Sousa, R. Greil, . 2022. Transcriptomic profiling identifies CD8^+^ T cells in the brain of aged and alzheimer’s disease transgenic mice as tissue-resident memory T cells. J. Immunol. 209:1272–1285. 10.4049/jimmunol.2100737 36165202 PMC9515311

[bib2] Barczykowski, A.L., A.H. Foss, P.K. Duffner, L. Yan, and R.L. Carter. 2012. Death rates in the U.S. due to Krabbe disease and related leukodystrophy and lysosomal storage diseases. Am. J. Med. Genet. A. 158A:2835–2842. 10.1002/ajmg.a.35624 22991292

[bib3] Becht, E., L. McInnes, J. Healy, C.A. Dutertre, I.W.H. Kwok, L.G. Ng, F. Ginhoux, and E.W. Newell. 2018. Dimensionality reduction for visualizing single-cell data using UMAP. Nat. Biotechnol. 10.1038/nbt.4314 30531897

[bib4] Bradbury, A.M., J.H. Bagel, X. Jiang, G.P. Swain, M.L. Prociuk, C.A. Fitzgerald, P.A. O’Donnell, K.G. Braund, D.S. Ory, and C.H. Vite. 2016. Clinical, electrophysiological, and biochemical markers of peripheral and central nervous system disease in canine globoid cell leukodystrophy (Krabbe’s disease). J. Neurosci. Res. 94:1007–1017. 10.1002/jnr.23838 27638585 PMC5027978

[bib5] Béchet, S., S.A. O’Sullivan, J. Yssel, S.G. Fagan, and K.K. Dev. 2020. Fingolimod rescues demyelination in a mouse model of Krabbe’s disease. J. Neurosci. 40:3104–3118. 10.1523/JNEUROSCI.2346-19.2020 32127495 PMC7141882

[bib6] Campisi, L., S. Chizari, J.S.Y. Ho, A. Gromova, F.J. Arnold, L. Mosca, X. Mei, Y. Fstkchyan, D. Torre, C. Beharry, . 2022. Clonally expanded CD8 T cells characterize amyotrophic lateral sclerosis-4. Nature. 606:945–952. 10.1038/s41586-022-04844-5 35732742 PMC10089623

[bib7] Corado, C.R., J. Pinkstaff, X. Jiang, E.M. Galban, S.J. Fisher, O. Scholler, C. Russell, J.H. Bagel, P.A. ODonnell, D.S. Ory, . 2020. Cerebrospinal fluid and serum glycosphingolipid biomarkers in canine globoid cell leukodystrophy (Krabbe Disease). Mol. Cell. Neurosci. 102:103451. 10.1016/j.mcn.2019.103451 31794880 PMC7032565

[bib8] Escolar, M.L., M.D. Poe, J.M. Provenzale, K.C. Richards, J. Allison, S. Wood, D.A. Wenger, D. Pietryga, D. Wall, M. Champagne, . 2005. Transplantation of umbilical-cord blood in babies with infantile Krabbe’s disease. N. Engl. J. Med. 352:2069–2081. 10.1056/NEJMoa042604 15901860

[bib9] Escolar, M.L., T. West, A. Dallavecchia, M.D. Poe, and K. LaPoint. 2016. Clinical management of Krabbe disease. J. Neurosci. Res. 94:1118–1125. 10.1002/jnr.23891 27638597

[bib10] Evans, F.L., M. Dittmer, A.G. de la Fuente, and D.C. Fitzgerald. 2019. Protective and regenerative roles of T cells in central nervous system disorders. Front. Immunol. 10:2171. 10.3389/fimmu.2019.02171 31572381 PMC6751344

[bib11] Feltri, M.L., N.I. Weinstock, J. Favret, N. Dhimal, L. Wrabetz, and D. Shin. 2021. Mechanisms of demyelination and neurodegeneration in globoid cell leukodystrophy. Glia. 69:2309–2331. 10.1002/glia.24008 33851745 PMC8502241

[bib12] Gate, D., N. Saligrama, O. Leventhal, A.C. Yang, M.S. Unger, J. Middeldorp, K. Chen, B. Lehallier, D. Channappa, M.B. De Los Santos, . 2020. Clonally expanded CD8 T cells patrol the cerebrospinal fluid in Alzheimer’s disease. Nature. 577:399–404. 10.1038/s41586-019-1895-7 31915375 PMC7445078

[bib13] Gross, C.C., C. Meyer, U. Bhatia, L. Yshii, I. Kleffner, J. Bauer, A.R. Tröscher, A. Schulte-Mecklenbeck, S. Herich, T. Schneider-Hohendorf, . 2019. CD8^+^ T cell-mediated endotheliopathy is a targetable mechanism of neuro-inflammation in Susac syndrome. Nat. Commun. 10:5779. 10.1038/s41467-019-13593-5 31852955 PMC6920411

[bib14] He, Y., K. Peng, R. Li, Z. Zhang, L. Pan, T. Zhang, A. Lin, R. Hong, Z. Nie, Q. Guan, and L. Jin. 2022. Changes of T lymphocyte subpopulations and their roles in predicting the risk of Parkinson’s disease. J. Neurol. 269:5368–5381. 10.1007/s00415-022-11190-z 35608657 PMC9467943

[bib15] Heller, G.J., M.S. Marshall, Y. Issa, J.N. Marshall, D. Nguyen, E. Rue, K.C. Pathmasiri, M.S. Domowicz, R.B. van Breemen, L.M. Tai, . 2021. Waning efficacy in a long-term AAV-mediated gene therapy study in the murine model of Krabbe disease. Mol. Ther. 29:1883–1902. 10.1016/j.ymthe.2021.01.026 33508430 PMC8116612

[bib16] Hordeaux, J., B.A. Jeffrey, J. Jian, G.R. Choudhury, K. Michalson, T.W. Mitchell, E.L. Buza, J. Chichester, C. Dyer, J. Bagel, . 2022. Efficacy and safety of a Krabbe disease gene therapy. Hum. Gene Ther. 33:499–517. 10.1089/hum.2021.245 35333110 PMC9142772

[bib17] Iacono, D., S. Koga, H. Peng, A. Manavalan, J. Daiker, M. Castanedes-Casey, N.B. Martin, A.R. Herdt, M.H. Gelb, D.W. Dickson, and C.W. Lee. 2022. Galactosylceramidase deficiency and pathological abnormalities in cerebral white matter of Krabbe disease. Neurobiol. Dis. 174:105862. 10.1016/j.nbd.2022.105862 36113749 PMC10474820

[bib18] Itoh, M., M. Hayashi, Y. Fujioka, K. Nagashima, Y. Morimatsu, and H. Matsuyama. 2002. Immunohistological study of globoid cell leukodystrophy. Brain Dev.. 24:284–290. 10.1016/S0387-7604(02)00057-8 12142065

[bib19] Kirby, L., J. Jin, J.G. Cardona, M.D. Smith, K.A. Martin, J. Wang, H. Strasburger, L. Herbst, M. Alexis, J. Karnell, . 2019. Oligodendrocyte precursor cells present antigen and are cytotoxic targets in inflammatory demyelination. Nat. Commun. 10:3887. 10.1038/s41467-019-11638-3 31467299 PMC6715717

[bib20] Korsunsky, I., N. Millard, J. Fan, K. Slowikowski, F. Zhang, K. Wei, Y. Baglaenko, M. Brenner, P.R. Loh, and S. Raychaudhuri. 2019. Fast, sensitive and accurate integration of single-cell data with Harmony. Nat. Methods. 16:1289–1296. 10.1038/s41592-019-0619-0 31740819 PMC6884693

[bib21] Krysko, K.M., J. Graves, M. Rensel, B. Weinstock-Guttman, G. Aaen, L. Benson, T. Chitnis, M. Gorman, M. Goyal, L. Krupp, . 2018. Use of newer disease-modifying therapies in pediatric multiple sclerosis in the US. Neurology. 91:e1778–e1787. 10.1212/WNL.0000000000006471 30333163 PMC6251604

[bib22] Kvistad, S.A.S., J. Burman, A.K. Lehmann, A. Tolf, C. Zjukovskaja, G.K. Melve, L. Bø, and Ø. Torkildsen. 2022. Impact of previous disease-modifying treatment on safety and efficacy in patients with MS treated with AHSCT. J. Neurol. Neurosurg. Psychiatry. 93:844–848. 10.1136/jnnp-2022-328797 35508373 PMC9304086

[bib23] Maloveska, M., J. Danko, E. Petrovova, L. Kresakova, K. Vdoviakova, A. Michalicova, A. Kovac, V. Cubinkova, and D. Cizkova. 2018. Dynamics of Evans blue clearance from cerebrospinal fluid into meningeal lymphatic vessels and deep cervical lymph nodes. Neurol. Res. 40:372–380. 10.1080/01616412.2018.1446282 29619904

[bib24] Marshall, M.S., Y. Issa, B. Jakubauskas, M. Stoskute, V. Elackattu, J.N. Marshall, W. Bogue, D. Nguyen, Z. Hauck, E. Rue, . 2018. Long-term improvement of neurological signs and metabolic dysfunction in a mouse model of Krabbe’s disease after global gene therapy. Mol. Ther. 26:874–889. 10.1016/j.ymthe.2018.01.009 29433937 PMC5910889

[bib25] Matsushima, G.K., M. Taniike, L.H. Glimcher, M.J. Grusby, J.A. Frelinger, K. Suzuki, and J.P. Ting. 1994. Absence of MHC class II molecules reduces CNS demyelination, microglial/macrophage infiltration, and twitching in murine globoid cell leukodystrophy. Cell. 78:645–656. 10.1016/0092-8674(94)90529-0 8069913

[bib26] Mi, H., D. Ebert, A. Muruganujan, C. Mills, L.P. Albou, T. Mushayamaha, and P.D. Thomas. 2021. PANTHER version 16: A revised family classification, tree-based classification tool, enhancer regions and extensive API. Nucleic Acids Res. 49:D394–D403. 10.1093/nar/gkaa1106 33290554 PMC7778891

[bib27] Mi, H., and P. Thomas. 2009. PANTHER pathway: An ontology-based pathway database coupled with data analysis tools. Methods Mol. Biol. 563:123–140. 10.1007/978-1-60761-175-2_7 19597783 PMC6608593

[bib28] Mohebiany, A.N., N.S. Ramphal, K. Karram, G. Di Liberto, T. Novkovic, M. Klein, F. Marini, M. Kreutzfeldt, F. Härtner, S.M. Lacher, . 2020. Microglial A20 protects the brain from CD8 T-Cell-Mediated immunopathology. Cell Rep. 30:1585–1597.e6. 10.1016/j.celrep.2019.12.097 32023471

[bib29] Nicaise, A.M., E.R. Bongarzone, and S.J. Crocker. 2016. A microglial hypothesis of globoid cell leukodystrophy pathology. J. Neurosci. Res. 94:1049–1061. 10.1002/jnr.23773 27638591 PMC5027969

[bib30] Ohno, M., A. Komiyama, P.M. Martin, and K. Suzuki. 1993. MHC class II antigen expression and T-cell infiltration in the demyelinating CNS and PNS of the twitcher mouse. Brain Res. 625:186–196. 10.1016/0006-8993(93)91058-Z 8275302

[bib31] Potter, G.B., and M.A. Petryniak. 2016. Neuroimmune mechanisms in Krabbe’s disease. J. Neurosci. Res. 94:1341–1348. 10.1002/jnr.23804 27638616 PMC5129482

[bib32] Potter, G.B., M. Santos, M.T. Davisson, D.H. Rowitch, D.L. Marks, E.R. Bongarzone, and M.A. Petryniak. 2013. Missense mutation in mouse GALC mimics human gene defect and offers new insights into Krabbe disease. Hum. Mol. Genet. 22:3397–3414. 10.1093/hmg/ddt190 23620143 PMC3736866

[bib33] Rentzos, M., E. Evangelopoulos, E. Sereti, V. Zouvelou, S. Marmara, T. Alexakis, and I. Evdokimidis. 2012. Alterations of T cell subsets in ALS: A systemic immune activation?. Acta Neurol. Scand. 125:260–264. 10.1111/j.1600-0404.2011.01528.x 21651502

[bib34] Santambrogio, S., A. Ricca, C. Maderna, A. Ieraci, M. Aureli, S. Sonnino, W. Kulik, P. Aimar, L. Bonfanti, S. Martino, and A. Gritti. 2012. The galactocerebrosidase enzyme contributes to maintain a functional neurogenic niche during early post-natal CNS development. Hum. Mol. Genet. 21:4732–4750. 10.1093/hmg/dds313 22859505

[bib35] Satija, R., J.A. Farrell, D. Gennert, A.F. Schier, and A. Regev. 2015. Spatial reconstruction of single-cell gene expression data. Nat. Biotechnol. 33:495–502. 10.1038/nbt.3192 25867923 PMC4430369

[bib36] Schrier Vergano, S.A., S. Kanungo, and G. Arnold. 2022. Making decisions about Krabbe disease newborn screening. Pediatrics. 149:e2021053175. 10.1542/peds.2021-053175 35229104

[bib37] Steinman, L., and S.S. Zamvil. 2006. How to successfully apply animal studies in experimental allergic encephalomyelitis to research on multiple sclerosis. Ann. Neurol. 60:12–21. 10.1002/ana.20913 16802293

[bib38] Suzuki, K., and M. Taniike. 1995. Murine model of genetic demyelinating disease: The twitcher mouse. Microsc. Res. Tech. 32:204–214. 10.1002/jemt.1070320304 8527855

[bib39] Szabo, P.A., H.M. Levitin, M. Miron, M.E. Snyder, T. Senda, J. Yuan, Y.L. Cheng, E.C. Bush, P. Dogra, P. Thapa, . 2019. Single-cell transcriptomics of human T cells reveals tissue and activation signatures in health and disease. Nat. Commun. 10:4706. 10.1038/s41467-019-12464-3 31624246 PMC6797728

[bib40] Taniike, M., J.R. Marcus, B. Popko, and K. Suzuki. 1997. Expression of major histocompatibility complex class I antigens in the demyelinating twitcher CNS and PNS. J. Neurosci. Res. 47:539–546. 10.1002/(SICI)1097-4547(19970301)47:5<539::AID-JNR9>3.0.CO;2-I 9067863

[bib41] Traag, V.A., L. Waltman, and N.J. van Eck. 2019. From louvain to leiden: Guaranteeing well-connected communities. Sci. Rep. 9:5233. 10.1038/s41598-019-41695-z 30914743 PMC6435756

[bib42] Wagner, C.A., P.J. Roqué, T.R. Mileur, D. Liggitt, and J.M. Goverman. 2020. Myelin-specific CD8+ T cells exacerbate brain inflammation in CNS autoimmunity. J. Clin. Invest. 130:203–213. 10.1172/JCI132531 31573979 PMC6934187

[bib43] Wasserstein, M.P., J.J. Orsini, A. Goldenberg, M. Caggana, P.A. Levy, M. Breilyn, and M.H. Gelb. 2021. The future of newborn screening for lysosomal disorders. Neurosci. Lett. 760:136080. 10.1016/j.neulet.2021.136080 34166724 PMC10387443

[bib44] Weinstock, N.I., D. Shin, N. Dhimal, X. Hong, E.E. Irons, N.J. Silvestri, C.B. Reed, D. Nguyen, O. Sampson, Y.C. Cheng, . 2020. Macrophages expressing GALC improve peripheral Krabbe disease by a mechanism independent of cross-correction. Neuron. 107:65–81.e9. 10.1016/j.neuron.2020.03.031 32375064 PMC7924901

[bib45] Wenger, D.A., P. Luzi. 2020. Krabbe disease: globoid cell leukodystrophy. In Rosenberg's molecular and genetic basis of neurological and psychiatric disease. Sixth edition. Academic Press, Cambridge. 481–491.

[bib46] Williams-Gray, C.H., R.S. Wijeyekoon, K.M. Scott, S. Hayat, R.A. Barker, and J.L. Jones. 2018. Abnormalities of age-related T cell senescence in Parkinson’s disease. J. Neuroinflammation. 15:166. 10.1186/s12974-018-1206-5 29807534 PMC5972443

[bib47] Willis, C.M., A.M. Nicaise, A. Menoret, J.K. Ryu, A.S. Mendiola, E.R. Jellison, M.I. Givogri, D.K. Han, E.R. Bongarzone, K. Akassoglou, . 2019. Extracellular vesicle fibrinogen induces encephalitogenic CD8+ T cells in a mouse model of multiple sclerosis. Proc. Natl. Acad. Sci. USA. 116:10488–10493. 10.1073/pnas.1816911116 31068461 PMC6535008

[bib48] Wu, Y.P., J. Matsuda, A. Kubota, K. Suzuki, and K. Suzuki. 2000. Infiltration of hematogenous lineage cells into the demyelinating central nervous system of twitcher mice. J. Neuropathol. Exp. Neurol. 59:628–639. 10.1093/jnen/59.7.628 10901235

[bib49] Wu, Y.P., E.J. McMahon, J. Matsuda, K. Suzuki, G.K. Matsushima, and K. Suzuki. 2001. Expression of immune-related molecules is downregulated in twitcher mice following bone marrow transplantation. J. Neuropathol. Exp. Neurol. 60:1062–1074. 10.1093/jnen/60.11.1062 11706936

[bib50] Yuan, J., H.M. Levitin, V. Frattini, E.C. Bush, D.M. Boyett, J. Samanamud, M. Ceccarelli, A. Dovas, G. Zanazzi, P. Canoll, . 2018. Single-cell transcriptome analysis of lineage diversity in high-grade glioma. Genome Med. 10:57. 10.1186/s13073-018-0567-9 30041684 PMC6058390

[bib51] Zhang, X., Y. Lan, J. Xu, F. Quan, E. Zhao, C. Deng, T. Luo, L. Xu, G. Liao, M. Yan, . 2019. CellMarker: A manually curated resource of cell markers in human and mouse. Nucleic Acids Res. 47:D721–D728. 10.1093/nar/gky900 30289549 PMC6323899

[bib52] Zheng, G.X., J.M. Terry, P. Belgrader, P. Ryvkin, Z.W. Bent, R. Wilson, S.B. Ziraldo, T.D. Wheeler, G.P. McDermott, J. Zhu, . 2017. Massively parallel digital transcriptional profiling of single cells. Nat. Commun. 8:14049. 10.1038/ncomms14049 28091601 PMC5241818

