# Supplementary material for: CD8+ T cell depletion prevents neuropathology in a mouse model of globoid cell leukodystrophy
Source: J Exp Med. 2023 Jun 13;220(9):e20221862. doi: 10.1084/jem.20221862 (PMC10266545; doi:10.1084/jem.20221862)
Supplement: Table S1 — lists antibodies. [file JEM_20221862_TableS1.docx]

Table S1. List of Antibodies

| **Antibody** | **Concentration** | **Source** | **Clone** |
| --- | --- | --- | --- |
| Flow Cytometry |  |  |  |
| CD45 (APC) | 1:200 | BioLegend | 30-F11 |
| CD3 (PE/Cy7) | 1:200 | BioLegend | 145-2C11 |
| CD4 (PE) | 1:200 | BioLegend | GK1.5 |
| CD4 (PE-AF610) | 1:200 | eBiosciences | RM4-5 |
| CD8α (APC/Cy7)) | 1:400 | BioLegend | 53-6.7 |
| CD44 (V500) | 1:400 | BD Horizon | IM7 |
| CD62L (PE-594) | 1:200 | BD Horizon | MEL-14 |
| Vβ2 (PE) | 1:200 | BioLegend | B20.6 |
| Vβ2 (FITC) | 1:200 | BD Horizon | B20.6 |
| Vβ3 (BV510) | 1:200 | BD Horizon | KJ25 |
| Vβ4 (BV711) | 1:200 | BD Horizon | KT4 |
| Vβ5.1/5.2 (Biotin) | 1:100 | PharMigen | MR9-4 |
| Vβ6 (APC) | 1:200 | eBiosciences | RR4-7 |
| Vβ7 (PE) | 1:200 | BD Horizon | TR310 |
| Vβ8.1/8.2 (PE) | 1:200 | BD Horizon | 1B3.3 |
| Vβ8.3 (FITC) | 1:200 | BD Horizon | MR5-2 |
| Vβ9 (FITC) | 1:200 | BD Horizon | MR10-2 |
| Vβ10 (BV605) | 1:200 | BD Horizon | B21.5 |
| Vβ11 (AF647) | 1:200 | BioLegend | KT11 |
| Vβ12 (BV711) | 1:200 | BD Horizon | MR111-1 |
| Vβ13 (PE) | 1:200 | BD Horizon | MR12-3 |
| Vβ13 (FITC) | 1:200 | BD Horizon | MR12-3 |
| Vβ14 (Biotin) | 1:200 | BD Horizon | 14-2 |
| Vβ17 (BV510) | 1:200 | BD Horizon | KJ23 |
| IFNγ (PE-Dazzle) | 1:200 | BioLegend | XMG1.2 |
| TNFα (PE) | 1:200 | BioLegend | MP6-XT22 |
| IL6 (PE-EF710) | 1:200 | eBiosciences | MP5-20F3 |
| DAPI | 1:10,000 | Roche | - |
| Zombie UV Live/Dead | 1:1000 | BioLegend | - |
| Immunohistochemistry |  |  |  |
| MBP | 1:200 | Chemicon | - |
| CD8⍺ | 1:500 | BioXCell | - |
| CD8 | 1:100 | Abcam | - |
| GFA (conjugated to Cy3) | 1:400 | Sigma-Aldrich | - |
| IBA1 | 1:250 | Wako | - |
| DAPI | 1:500 | Roche | - |
| GZMB | 1:50 | Invitrogen | - |
| ISG15 | 1:500 | Invitrogen | - |
| CXCR6 | 1:500 | Invitrogen | - |
| TNFa | 1:50 | Invitrogen | - |
| IL6 | 1:100 | Invitrogen | - |
| In vivo |  |  |  |
| Anti-mouse CD8⍺ | 300µg, i.p. | BioXCell | 2.43 |
| Anti-mouse IgG2a Isotype | 300µg, i.p. | BioXCell | C1.18.4 |
