# Supplementary material for: CD8+ T cell depletion prevents neuropathology in a mouse model of globoid cell leukodystrophy
Source: J Exp Med. 2023 Jun 13;220(9):e20221862. doi: 10.1084/jem.20221862 (PMC10266545; doi:10.1084/jem.20221862)
Supplement: Table S2 — shows RT-qPCR primers. [file JEM_20221862_TableS2.docx]

Table S2. RT-qPCR Primers

| **Target** | **Forward (5’-3’)** | **Reverse (5’-3’)** | **Species** |
| --- | --- | --- | --- |
| GFAP | TCCTGGAACAGCAAAACAAG | CAGCCTCAGGTTGGTTTCAT | Mouse |
| IBA1 | GTCCTTGAAGCGAATGCTGG | CATTCTCAAGATGGCAGATC | Mouse |
| CD86 | ACGATGGACCCCAGATGCACCA | GCGTCTCCACGGAAACAGCA | Mouse |
| Β-actin | CTGGCTCCTAGCACCATGAA | CGCAGCYCAGTAACAGTCCG | Mouse |
